# A New Prevalent Densovirus Discovered in *Acari*. Insight from Metagenomics in Viral Communities Associated with Two-Spotted Mite (*Tetranychus urticae*) Populations

**DOI:** 10.3390/v11030233

**Published:** 2019-03-07

**Authors:** Sarah François, Doriane Mutuel, Alison B. Duncan, Leonor R. Rodrigues, Celya Danzelle, Sophie Lefevre, Inês Santos, Marie Frayssinet, Emmanuel Fernandez, Denis Filloux, Philippe Roumagnac, Rémy Froissart, Mylène Ogliastro

**Affiliations:** 1 DGIMI, Univ Montpellier, INRA, F-34095 Montpellier, France; sarah.francois.dgimi@gmail.com (S.F.); Doriane.mutuel@inra.fr (D.M.); celya.danzelle@icloud.com (C.D.); marie.frayssinet@inra.fr (M.F.); 2 ISEM, Univ Montpellier, CNRS, IRD, EPHE, F-34095 Montpellier, France; alison.duncan@umontpellier.fr (A.B.D.); sophie.lefevre@umontpellier.fr (S.L.); 3 Centre for Ecology, Evolution and Environmental Changes (cE3c), Univ Lisbon, Faculty of Science, P-1749016 Lisbon, Portugal; leonor.rodrigues89@gmail.com (L.R.R.); inesflsantos@gmail.com (I.S.); 4 CIRAD, UMR BGPI, F-34398 Montpellier, France; emmanuel.fernandez@cirad.fr (E.F.); denis.filloux@cirad.fr (D.F.); philippe.roumagnac@cirad.fr (P.R.); 5 BGPI, Univ Montpellier, INRA, CIRAD, Montpellier SupAgro, F-34398 Montpellier, France; 6 MIVEGEC, Univ Montpellier, CNRS, IRD, F-34394 Montpellier, France; remy.froissart@ird.fr

**Keywords:** parvovirus, viral metagenomics, virus diversity, virus phylogeny, agricultural pests, arthropod, mite, viral communities, viral ecology

## Abstract

Viral metagenomics and high throughput sequence mining have revealed unexpected diversity, and the potential presence, of parvoviruses in animals from all phyla. Among arthropods, this diversity highlights the poor knowledge that we have regarding the evolutionary history of densoviruses. The aim of this study was to explore densovirus diversity in a small arthropod pest belonging to *Acari*, the two-spotted spider mite *Tetranychus urticae*, while using viral metagenomics based on virus-enrichment. Here, we present the viromes obtained from *T. urticae* laboratory populations made of contigs that are attributed to nine new potential viral species, including the complete sequence of a novel densovirus. The genome of this densovirus has an ambisens genomic organization and an unusually compact size with particularly small non-structural proteins and a predicted major capsid protein that lacks the typical PLA2 motif that is common to all ambidensoviruses described so far. In addition, we showed that this new densovirus had a wide prevalence across populations of mite species tested and a genomic diversity that likely correlates with the host phylogeny. In particular, we observed a low densovirus genomic diversity between the laboratory and natural populations, which suggests that virus within-species evolution is probably slower than initially thought. Lastly, we showed that this novel densovirus can be inoculated to the host plant following feeding by infected mites, and circulate through the plant vascular system. These findings offer new insights into densovirus prevalence, evolution, and ecology.

## 1. Introduction

Arthropod-infecting parvoviruses, termed densoviruses, have been mostly discovered with a pathology-driven approach, which probably explains the relatively poor number of viral species (i.e., less than 60) that the International Committee for the Taxonomy of Viruses (ICTV) have referenced so far, and the strong bias that exists towards viruses infecting arthropods of health or economic importance [1].

Breakthrough techniques in viral metagenomics and mining high throughput sequencing (HGS) datasets have highlighted the extraordinary diversity and persistence of parvoviruses and parvovirus-related sequences in an unexpected array of animals, including invertebrates [2,3,4]. These discoveries support the now generally accepted view that viruses are integral components of the microbiome and participate in the functioning of ecosystems of differing size and complexity, i.e., an individual organism or a population of organisms. In the context of the general loss of biodiversity, which is particularly worrisome among arthropods, the question of the prevalence and diversity of their associated microorganisms, including viruses, is of particular interest [5].

Mites and ticks are small arachnids that belong to the *Acari* sub-class that are mostly known for their detrimental impact on human, animal, and plant health [6]. While ticks represent a relatively small number of taxa (around 900 species), all share a parasitic, blood-feeding alimentary regime; mites are extraordinarily diversified (more than 40,000 species) and exhibit a large diversity of lifestyles, including plant feeders, mite-predators, or arthropod ectoparasites, with only a few species presenting direct threats to plant or animal health. Among harmful mites, the two-spotted spider mite (*Tetranychus urticae*) is an agricultural pest that can cause significant damage to more than 1100 species of food-producing or flower cultures [7,8]. Treatment against *T. urticae* with synthetic acaricides has resulted in one of the highest incidences of pesticide resistance being recorded in arthropods [9]. In this context, the development of alternative solutions to chemicals is strongly encouraged; one promising way could be to diversify the use of spider mites’ natural enemies, and particularly to include their pathogens.

Despite their environmental success and the threat that they represent for agriculture, due to their small size and the poor description of their associated pathologies, spider-mites have been long neglected by virologists. Only one non-occluded, rod-shaped virus, related to a baculovirus, has so far been described following infections in laboratory populations of mites [10,11]. More recently, viral metagenomics and database mining being applied to *Acari* was developed to explore the viral communities (so-called the viromes) that are associated with blood-feeding ticks [12,13,14,15]. These studies revealed an extraordinary wealth of viruses, including parvovirus related sequences, and they validated this approach for virus discovery in these small arthropods. A similar approach was also developed to analyze viruses that are associated with *Varroa destructor*, an ectoparasitic mite of honey bees that also transmits several viruses to the bees and are suspected to contribute to colony collapse [16,17]. This work revealed that several viruses from different families can be found in Varroa at the population level, including mite associated viruses that are common in arthropods (e.g., *Baculoviridae*, *Circoviridae*, *Dicistroviridae*, and *Iflaviridae*).

Herein, we used viral metagenomics and in depth sequencing of virion-associated nucleic acids (VANA) [18] to explore the viral communities that are associated with *T. urticae* from two laboratory populations of different geographic origins and rearing host plants. We found eight new putative virus species belonging to taxa that are associated with arthropods and one with fungi, with five of those viral sequences being shared by both mite populations. More specifically, we discovered that the most abundant reads found in both viromes correspond to a new densovirus and probably a new divergent species in the *Ambidensovirus* genus. RACE, PCR, and sequencing confirmed the genome of this new densovirus and the expression of the viral genes in mites. This new densovirus displayed a very compact genome, and the predicted sequence of its major capsid protein lacked the phospholipase A2 motif that is shared so far by all the members of this genus. This virus, initially named Tetranychus urticae-associated ambidensovirus (TuaDV), was also prevalent in different species of spider mites from laboratory populations worldwide and sampled from natural environments. Comparing TuaDV sequence diversity reveals that the virus is highly conserved within each species, but that there is distinct variation between species that are indicative of some virus-host coevolution [19,20]. Finally, we analyzed TuaDV transmission and found that infection can potentially occur via horizontal and vertical routes. In particular, we showed that mites, when feeding, could transfer viral particles to host plants, which could then be further circulated *in planta*, probably through the vascular system. This mechanism, already shown for the aphid densovirus MpDV, suggests that the plant may be used to spread infection to conspecifics and/or to other species [21].

These discoveries expand our knowledge of densovirus prevalence and diversity in an arthropod taxon that has long been neglected by virologists.

## 2. Materials and Methods

### 2.1. Spider-Mite Populations and Rearing

Two laboratory *T. urticae* populations from Portugal and France were first analyzed using the VANA metagenomics-based approach to identify the potential virus species. The first population, the so-called the Portuguese (P) population corresponds to a mix of *T. cinnabarinus* and *T. urticae* populations established at the University of Lisbon. This mix comprised a *T. cinnabarinus* population collected in January 2014 in Spain (Almeria) on rose plants (so-called Almeria population), and two *T. urticae* strains; the “London strain”, originating from the Vineland region, Ontario, Canada [8] and the “EtoxR” strain originating from Japan and maintained for five years in the laboratory at Bayer CropScience [22]. All of the populations and strains were maintained on bean plants in the laboratory. It should be noted that this mix was established before the distinction was made between *T. urticae* and *T. cinnabarinus* as possibly being separate species and not two morphs of the same species. The second population, so-called the French (F) population was reared in Montpellier and it also constitutes a mix of populations. The majority of these originate from a *T. urticae* collection in May 1994 in the Netherlands (NL, Pijnacker) from cucumber, which were subsequently transferred to Montpellier in 2007, at which time they were split into two populations (still maintained on cucumber plants). In 2011, these two populations were mixed, and subsequently transferred to either tomato, bean, or kept on cucumber plants (four populations being created per host plant type). Note that two of these populations (HH4 and HH3) that were transferred to bean in 2011 were used to characterize TuaDV transmission. A second batch of mites (hereafter called “Mix-Tu”) that were used to characterize transmission contained a mix of 11 different mite populations; eight collected in Portugal, two in Spain (including the population collected in Almeria mentioned above), and one in France. All of the laboratory populations were maintained at 25 °C with an 8: 16 Light: Dark (L:D) cycle.

Once the TuaDV virus was identified in the mixes, we measured its prevalence in spider mite populations by analyzing a total of twenty-seven populations from three species of mites (*T. urticae*, *T. cinnabarinus*, and *T. evansi*), out of which TuaDV from eleven populations were partially or fully sequenced (Table S1). Specifically, the mites came from rearing facilities in France (Nice-Valbonne), Greece (Crete), the Netherlands (Santpoort), Belgium (Ghent), and Portugal (Lisbon), and from natural populations across Portugal sampled in the summer of 2017.

### 2.2. Preparation of Viromes and Sequencing

The samples were made from pools of around 200 individual mites, from which viral particles were purified using the method that was described by Francois et al. [18]. Briefly, the mites were ground in HBSS buffer with beads using a tissue homogenizer. The homogenized extracts were filtered through a 0.45 µm filter and centrifuged at 148.000 × g for 2.5 h at 4 °C to concentrate viral particles. Next, non-encapsidated nucleic acids were eliminated by DNase and RNase digestion for 1.5 h at 37 °C, although some of the non-encapsidated nucleic acids and cellular contamination may remain. Encapsidated DNA and RNA were then extracted using Nucleospin 96 virus Core Kit (Macherey Nagel, Düren, Germany) and RNA was converted to cDNA using a 26 nt primer (Dodeca Linker) composed of a 14 nt linker linked at the 3’ end to N_12_. Double-stranded DNA was synthetized from single-stranded DNA using Large (Klenow) fragment DNA polymerase and the Dodeca Linker. Double-stranded DNA was further amplified using one 24 nt PCR multiplex identifier primer that was composed of the 14 nt linker used during the RT step linked at the 5’ end to a 10 nt tag that allowed for sample identification. The PCR products were cleaned with QIAquick PCR Purification kit (Qiagen, Courtaboeuf, France) and the libraries were sequenced on Illumina MiSeq pair-end 300 nt (Genewiz, South Plainfield, NJ, USA).

### 2.3. Bioinformatics Analyses and Database Screening

Demultiplexing was done with the agrep command-line tool to assign reads to the samples from which they originated [23]. Adaptors were removed and the reads were filtered for quality (q30 quality and read length >45 nt) using Cutadapt 1.9 [24]. The cleaned reads were assembled de novo into contigs using SPAdes 3.6.2 (k-mer lengths 21,33,55,77,125) [25] and mapping was performed on contigs of aligned clean reads with Bowtie 2.1.0 (options local very sensitive) [26]. Taxonomic assignment was achieved through searches against the NCBI RefSeq viral database and against the non-redundant (nr) GenBank database using BLASTx with an e-value cutoff of <10^−3^ [27]. Viral contigs were classified as viral operational taxonomic units (vOTU). The most abundant vOTUs were subsequently characterized using an arbitrary abundance cutoff of < 0.1% that was applied for each vOTU in all of the samples to remove the inter-sample contamination that occurred during the preparation and the sequencing of the samples. This abundance threshold was chosen to be twice above to the most abundant virus taxa that were found in the negative control (HBSS buffer). The predicted sequence of full-length viral proteins were aligned and compared with their closest related viruses (found in GenBank database) using MUSCLE 3.7 (16 iterations) [28], according to the species demarcation thresholds that were recommended within the online reports of the ICTV (www.ictv.global/report/parvoviridae).

For database screening, sequences from all viral contigs were used as queries, as well as the genomes contained in NCBI Viral genome database to perform BLASTn searches within the *T. urticae* genome: RefSeq genomic database (GCF_000239435.1, 641 sequences), WGS (CAEY00000000.1, 2035 sequences) and transcriptomes: EST (txid32264, 80855 sequences) and TSA (BioProject 78685, 9614 sequences; BioProject 78689, 17739 sequences; BioProject 6829 sequences), with and an e-value cutoff of <10^−3^.

### 2.4. Validation of the TuaDV Full-Length Genomic Sequence

To confirm the presence of TuaDV in mites and confirm its complete coding sequence, pools of mites (n~100) from several populations were tested using PCR. Total DNA was extracted with Wizard Genomic kit (Promega Corp., Madison, WI, USA) and then recovered in a final volume 100 µL (100–135 ng/µL). PCR was performed from 100 ng DNA with specific overlapping sets of primers covering the full length of the genome (Table S2) and while using the GoTaq reaction mix (Promega). The amplicons were sequenced with the Sanger method. To determine the extremities of the TuaDV genome, we performed 5’/3’ RACE with specifically designed primers (Table S2). Total RNA was extracted from pools of mites from the HH4 population with the RNeasy minikit (Qiagen). 3’ and 5’RACE PCR were performed using the 5’/3’ RACE kit, 2nd Generation (Roche, Germany), according to the manufacturer’s instructions.

### 2.5. Phylogenetic Analyses

The putative amino acid sequences of *Tetranychus* associated vOTUs were used for phylogenetic analyses. All of the ORFs were translated in silico using the ORF finder (cut off >300 nt, ATG start codon) on Geneious 1.7 [29] and aligned with the corresponding protein fragments of related viruses being deposited on the GenBank nr database using MUSCLE 3.7 (16 iterations) with default settings [28]. The aligned sequences were manually edited to remove gaps. Maximum likelihood phylogenetic trees were produced from these alignments using PhyML 3.1 [30,31] with substitution models being chosen as the best-fit using Prottest 2.4 [32]. One-thousand bootstrap replicates were used to assign the strength of support for branches. Trees were visualized with FigTree 1.4 (http://tree.bio.ed.ac.uk/software%20/figtree/). Outgroups were used when possible, otherwise the trees were mid-point rooted.

### 2.6. Densovirus Presence and Transmission

The prevalence of TuaDV was assayed from pools of mites from the different populations using PCR, as described above. PCR was run using the following conditions: 95 °C for 2 min, 25 cycles of 95 °C for 45 sec, 57 °C for 45 sec, 72 °C for 1 min, and then 72 °C for 5 min and two sets of specific primers (61F and 693R (NS) and 1125 and 1793R (VP)). Amplicons were run in an agarose gel and stained with ethidium bromide. We also tested for the within-population prevalence of TuaDV in the laboratory population HH4. Extracting DNA from 20 individual mites from this population achieved this and running PCRs, as described above, to test for the presence of the virus.

When considering the universal presence of the virus, a separate experiment tested whether *T. urticae* associated densovirus was vertically transmitted from mothers to their offspring. To do this, 10 females from the HH4 population were isolated on individual bean leaves placed on water saturated cotton, and left to lay eggs for two days at 25 °C with a 8: 16 L: D cycle. Each female laid eggs that were recovered individually with a clean pair of tweezers and transferred to a new individual leaf patch that was placed on water saturated cotton, allowed to hatch, and offspring to develop into adult. Offspring (*n* = 10) that became adults and their mother were individually tested for viral infection. Total DNA was extracted from each individual mite, as described above, and recovered in 25 µL (8–12 ng/µL per mite). PCR reactions were set as above and run with 40 cycles. Amplicons were run in an agarose gel and stained with ethidium bromide. The detection limit of the method is estimated ~10ng.

To test for TuaDV inoculation and circulation in the host plant, we placed 50 infected mites on an individual leaf of a whole bean plant (8–10 leaves stage). Paraffin jelly was placed at the base of the petiole of each leaf in order to prevent mites from dispersing from the infested leaf to other leaves on the plant. Five days later, the mites were carefully removed from the infected leaf (named L0) and two uninfected leaves from the same plant, one ~8 cm and the other ~20 cm away from the infected leaf, were collected (named L8 and L20, respectively). We performed six independent transmission experiments with the HH4, HH3, and Mix-Tu populations. Three leaves from six non-infested plants were similarly taken and processed as negative controls. Total DNA was extracted from infested plants and negative controls using DNeasy blood and tissue kit (Qiagen) and recovered in 100 µL (40–60 ng/µL for leaves) and 25 µL (15–25 ng/µL for mites). The TuaDV inoculated to plant leaves was assessed by PCR, as described above, while viral loads in mites and leaves was achieved by qPCR with a reaction mixture containing 15–20 ng and 100 ng of mites or plant DNA, respectively, and a pair of TuaDV specific primers (1125F and 1230R, Table S2) using the Sensifast PCR kit (Bioline, UK). qPCR was run on a Roche LC480 cycler (qPHD facility, Univ Montpellier) using the following conditions: 95 °C for 2 min, 25 cycles of 95 °C for 30 sec, 60 °C for 30 sec, 72 °C for 50 sec, and then 72 °C for 5 min, which resulted in a 78 bp amplicon from ORF2 (NS). As the viral genome of TuaDV remains to be cloned, PCR Standardization was achieved with a standard curve that was made with the serial dilution of DNA of the Junonia coenia ambidensovirus (JcDV) as a proxy.

Statistical analyses were performed with the JMP® Version 11 software (SAS Institute Inc., Cary, NC, USA). The analysis of viral load (ng µL^−1^) within each leaf was performed using a general linear model with leaves (infected, uninfected 8cm or 20 cm away) and mite populations (HH3, HH4 and Mix-Tu) being included in the model as fixed factors. The plant from where the leaves were taken was included in the model as a random factor nested within population.

Accession numbers: The virus sequences reported herein have been deposited in the GenBank database under accession numbers MK533146 to MK533158 and MK543949.

## 3. Results

### 3.1. Overview of the Spider Mite Virome

To explore the viral diversity of *T. urticae* populations, we prepared viral particles according to the VANA protocol, from two laboratory populations from France (Montpellier, F population) and Portugal (Lisbon, P population), having different geographical origins and rearing history. Pools of mites from both of the populations were processed for virus-enrichment (VANA) metagenomics and high throughput sequencing. A total of 680,600 cleaned reads were obtained, including 219,982 reads from the French (F) population and 460 618 reads from the Portuguese (P) population.

**Figure 1:**
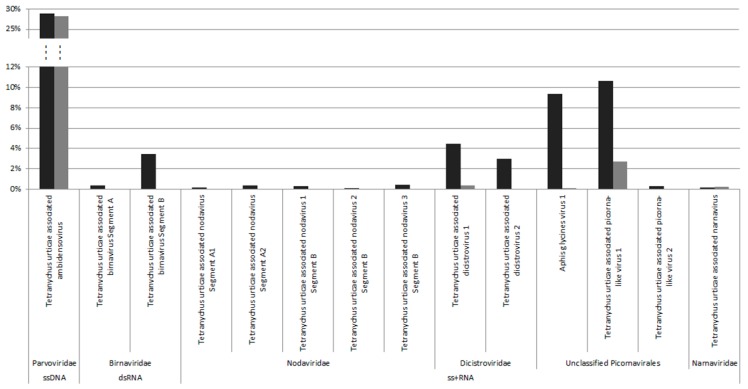
Relative abundance of putative viral species in the viromes of Portuguese (black) and French (grey) *T. urticae* colonies.

*De novo* assembly of the VANA reads in both populations obtained fourteen viral contigs (1.6 to 8.6 kb in length) (Table 1). Thirteen out of the fourteen contigs were mostly related to non-enveloped DNA and RNA viruses belonging to clades infecting arthropods, including *Dicistroviridae*, *Parvoviridae*, *Birnaviridae, Nodaviridae*, and unclassified picornavirales. In addition, one viral contig (2481 nt) was similar to yeast and fungi-infecting viruses of the *Narnaviridae* family, which might come from environmental contamination (e.g., food), even though we cannot exclude its replication in spider mites [33]. While nine viral contigs were isolated in the P population only, five viral contigs were common to both of the populations (Figure 1), which was surprising given their different origins and rearing history.

**Figure 2:**
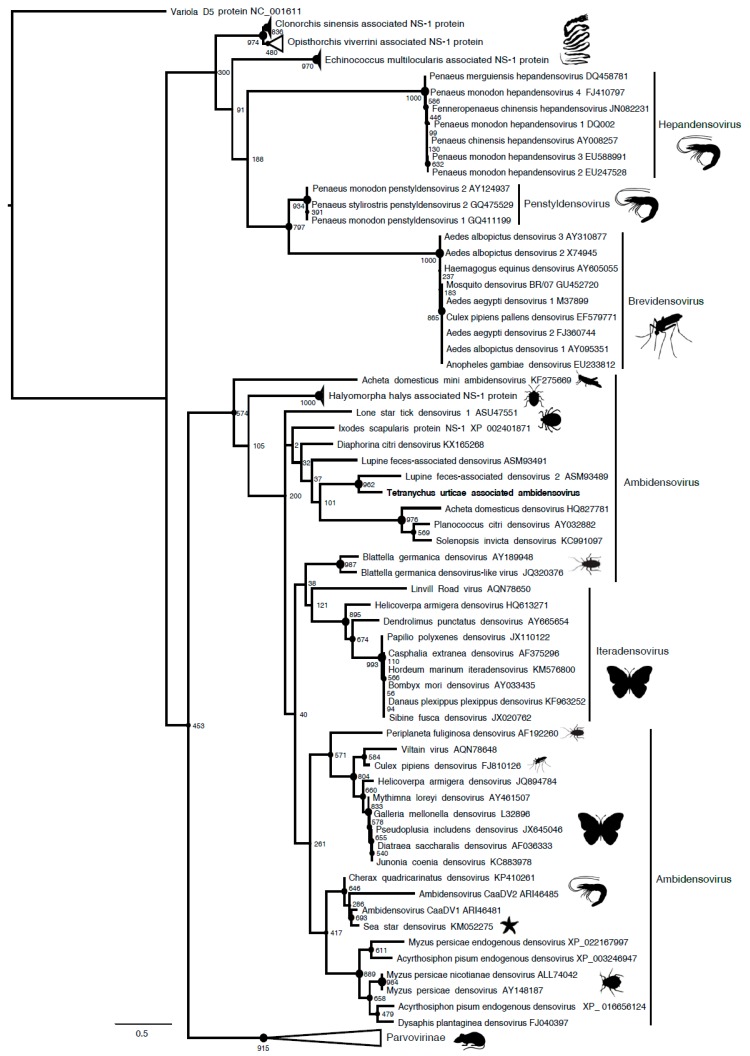

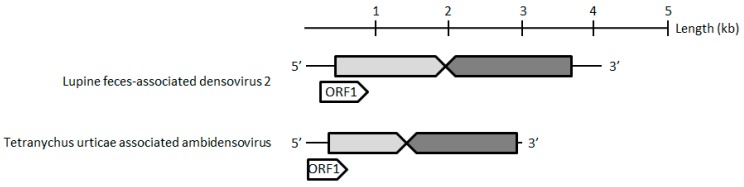
Maximum *Parvoviridae* likelihood phylogenetic tree based on a part of the NS1 protein containing the SF3 domain, including 105 parvovirus sequences and Tetranychus urticae-associated ambidensovirus (in bold). The alignment of 155 amino acids in length was produced using MUSCLE 3.7 (16 iterations) and was ungapped by hand. The tree was rooted with the SF3 domain of the Variola virus D5 protein. Bootstrap values are indicated at each node. Scale bar corresponds to amino acid substitutions per site. Genera of the *Parvoviridae* family are indicated in brackets. Genomic organization of Tetranychus urticae-associated ambidensovirus is also indicated. Grey arrows and rectangles: predicted open reading frames (ORF), Light grey: putative NS; dark grey: putative capsid protein (CP or VP). Arrow: complete ORF.

Among the five viral contigs that are common to both viromes, the one that is assigned to the *Parvoviridae* family was largely dominant in terms of reads abundance (>28% of viral reads in both populations), with the others being found at much lower frequencies (0.1% to 10.6%) (Figure 1). Sequence analysis and open reading frame (ORF) prediction showed that this parvovirus has an ambisense genomic organization with a predicted small Non Structural protein-1 (NS1) that shared 39% aa identity with the NS1 of its closest relative, the Lupine feces-associated densovirus 2, which was discovered from viral gut metagenomics of the iberian wolf and has no associated arthropod host so far (accession number: ASM93489). Accordingly, the phylogenetic tree that is based on the NS1 protein clustered this densovirus with Lupine feces-associated densovirus 2 among the *Ambidensovirus* genus (Figure 2). According to the new species demarcation threshold in the *Densovirinae* sub-family that was proposed by the ICTV (i.e., < 85% related by NS1 amino acid sequence identity [34]), this new densovirus might represent a new divergent species in the *Ambidensovirus* genus and the first densovirus isolated from *Acari* (Figure 2, Table 1). Hereafter, this virus is referred to as Tetranychus urticae-associated ambidensovirus (TuaDV).

**Figure 3:**
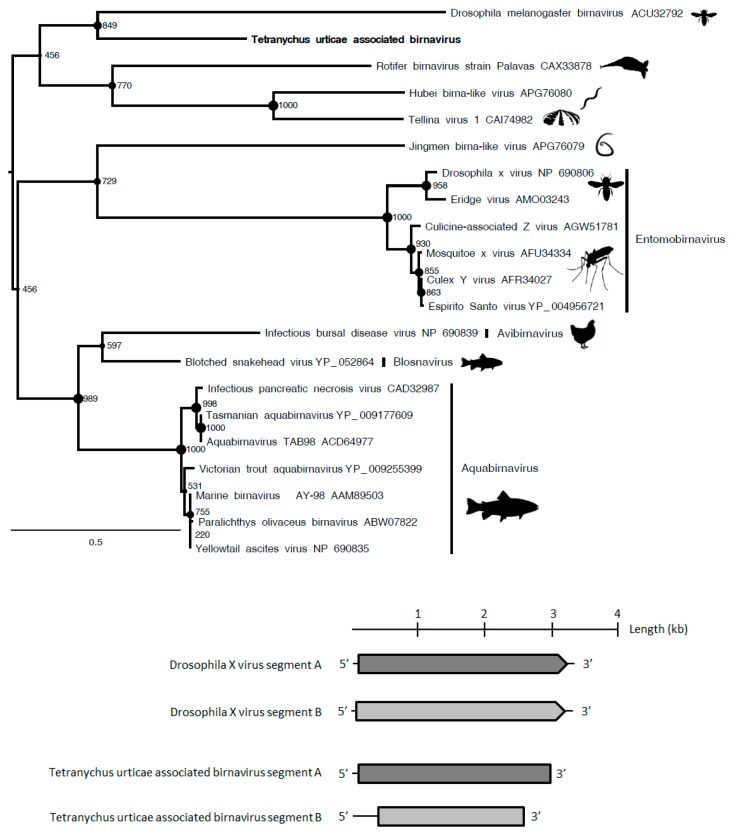
Maximum *Birnaviridae* likelihood phylogenetic tree based on part of the polyprotein, including 20 birnavirus species and Tetranychus urticae-associated birnavirus (in bold). The alignment of 602 amino acids in length was produced using MUSCLE 3.7 (16 iterations) and was ungapped by hand. The tree was mid-point rooted. Bootstrap values are indicated at each node. Scale bar corresponds to amino acid substitutions per site. Genera of the *Birnaviridae* family are indicated in brackets. Genomic organization of Tetranychus urticae-associated birnavirus is also indicated. Grey arrows and rectangles: predicted open reading frames (ORF), Light grey: putative NS; dark grey: putative capsid protein (CP). Arrow: complete ORF; Rectangle: truncated ORF.

Although the *Birnaviridae* family has been poorly investigated, the phylogenetic analyses showed that the polymerase protein of Tetranychus urticae-associated birnavirus that was found in *T. urticae* viromes (representing 3.8% of reads) clustered with Drosophila melanogaster birnavirus (Figure 3) within an unclassified lineage. The polymerase and capsid proteins of this putative novel entomobirnavirus also shared 30% aa identity with the Infectious bursal disease virus (accession number AAS10174.1) and 33% aa identity with the Blotched snakehead virus (accession number YP_052864.1), respectively. Therefore, this putative novel birnavirus could represent the first birnavirus isolated from Arachnids (Table 1).

**Figure 4:**
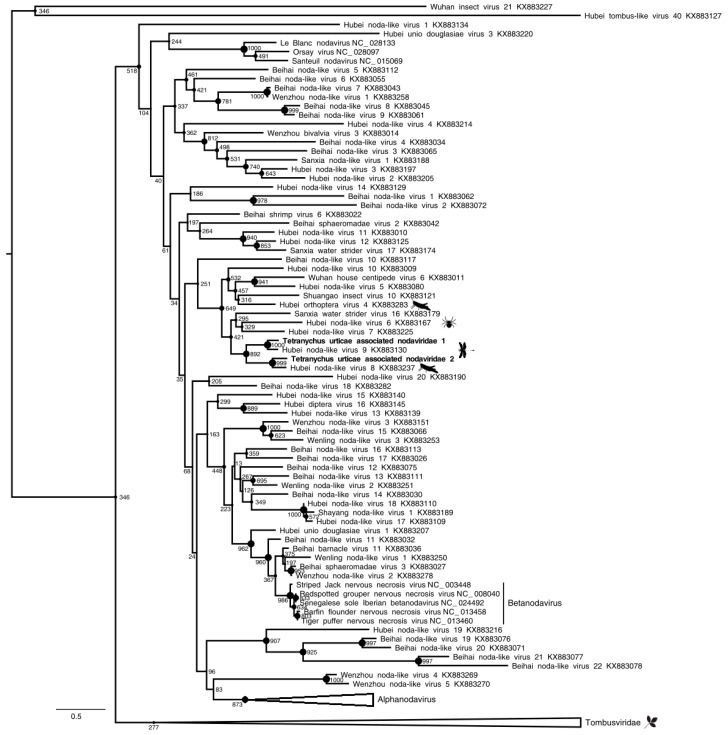

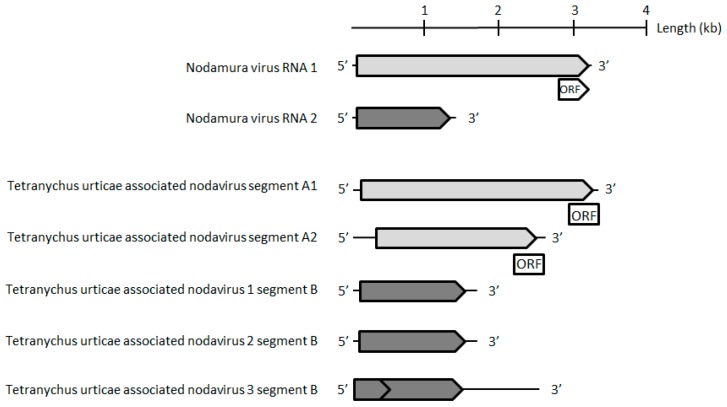
Maximum *Nodaviridae* likelihood phylogenetic tree based on part of the capsid protein, including 239 species and Tetranychus urticae-associated nodaviruses (in bold). The alignment of 229 amino acids in length was produced using MUSCLE 3.7 (16 iterations) and was ungapped by hand. The tree was mid-point rooted. Bootstrap values are indicated at each node. Scale bar corresponds to amino acid substitutions per site. Genomic organization of Tetranychus urticae-associated nodaviruses is also indicated. Grey arrows and rectangles: predicted open reading frames (ORF), Light grey: putative NS; dark grey: putative capsid protein (CP). Arrow: complete ORF; Rectangle: truncated ORF.

Regarding two of the three viral contigs clustering within the *Nodaviridae* family (Figure 4, Table 1), coding for the capsid protein, and found in the P population only (representing 1.35% of reads), they share up to 58% aa identity with the Hubei noda-like virus 9 capsid protein (accession number: YP_009337880.1), an unclassified RNA virus. According to the ICTV species demarcation threshold (<87% of capsid protein aa identity) and to the position of polymerase proteins in the phylogenetic tree, the nodaviruses that were found in *T. urticae* viromes may correspond to novel species-level lineages in a novel genus-level lineage in the *Nodaviridae* family (Figure 4, Table 1).

**Figure 5:**
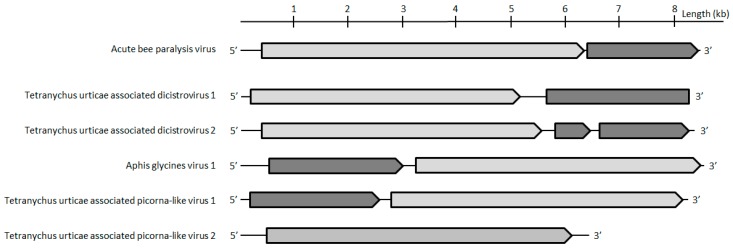
Maximum *Picornavirales* likelihood phylogenetic tree based on part of the polymerase protein, including 504 sequences and Tetranychus urticae-associated picornaviruses (in bold). The alignment of 386 amino acids in length was produced using MUSCLE 3.7 (16 iterations) and was ungapped by hand. The tree was mid-point rooted. Bootstrap values are indicated at each node, values <50% were discarded to not overload the tree. Scale bar corresponds to amino acid substitutions per site. Families of the *Picornavirales* order and genera of the *Dicistroviridae* family are indicated in brackets. Genomic organization of Tetranychus urticae-associated picornaviruses is also indicated. Grey arrows and rectangles: predicted open reading frames (ORF), Light grey: putative NS; dark grey: putative CP. Arrows: complete ORF.

Five contigs were assigned to the *Picornavirales* order. While two contigs clustered in the *Dicistroviridae* family (hereafter referred to as Tetranychus urticae-associated dicistrovirus 1 and 2 (Tuad1 and Tuad2), the other three clustered with the unclassified Picorna-like viruses. The capsids of Tuad1 and Tuad2 both share 20% aa identity with Beihai picorna-like virus 70(accession number APG78062.1; Table 1). Based on the current species demarcation criteria that are used by the ICTV *Dicistroviridae* study group (<90% of capsid protein identity with closest relatives) and the phylogenetic analyses bases on the conserved polymerase protein (Figure 5, Table 1), it is likely that both of the contigs could belong to two novel species-level lineages of the *Dicistroviridae* family. Interestingly, the CP of one of the three unclassified picorna-like viruses shared >99% aa identity with the unclassified picorna-like Aphis glycines virus 1 (Table 1). In addition, the proteins of the two unclassified picorna-like viruses (hereafter referred to as *Tetranychus urticae*, being associated picorna-like virus 1 and 2) share 53% to 75% aa identity with Aphis glycines virus 1 and Hubei picorna-like virus 80 (Table 1). Their phylogenetic trees showed that they might belong to a highly divergent lineage within the *Picornavirales* order (Figure 5). Moreover, Tetranychus urticae-associated picorna-like virus *1* and *2* could represent a new species according to species demarcation criteria that are defined by the ICTV (<90% of capsid protein identity with closest relatives) (Table 1).

**Figure 6:**
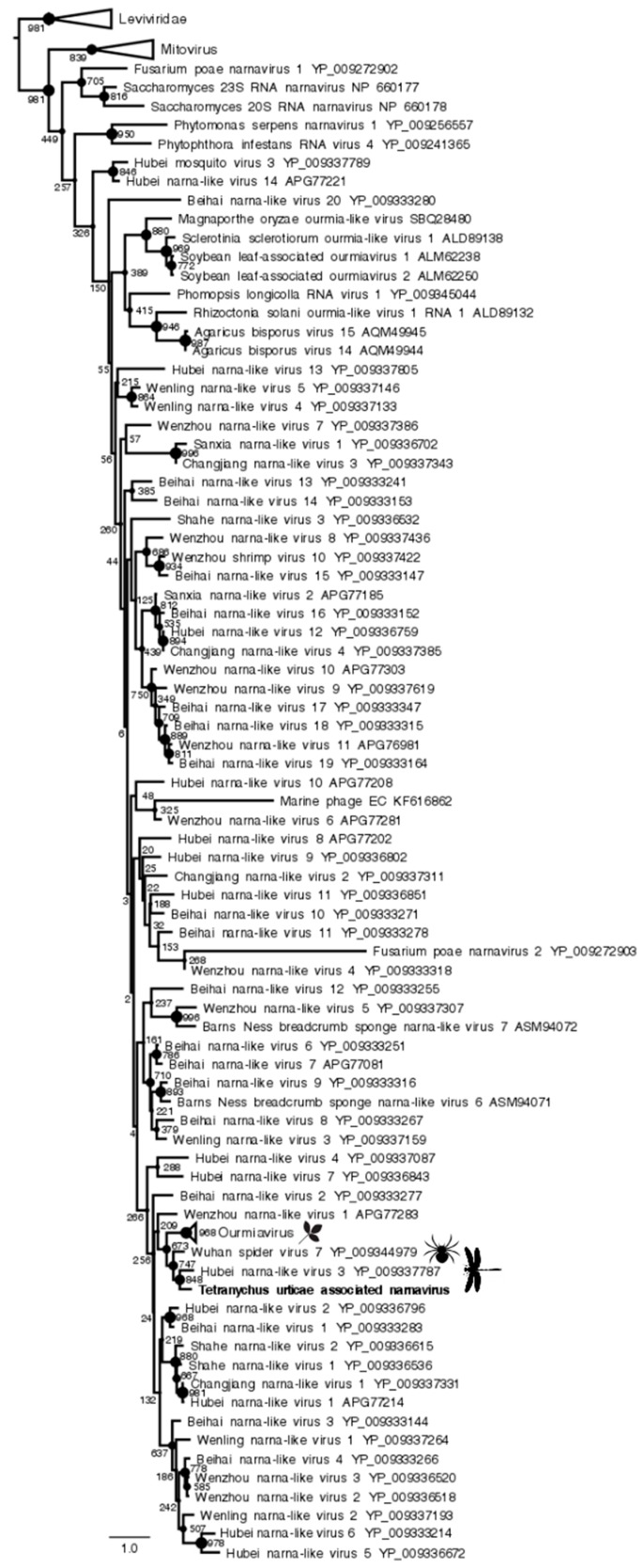

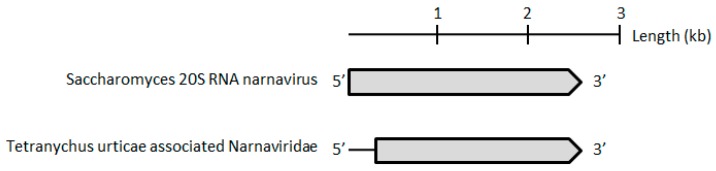
Maximum *Narnaviridae* likelihood phylogenetic tree based on part of the polymerase protein, including 129 species and Tetranychus urticae-associated narnavirus (in bold). The alignment of 121 amino acids in length was produced using MUSCLE 3.7 (16 iterations) and was ungapped by hand. Bootstrap values are indicated at each node. Scale bar corresponds to amino acid substitutions per site. Genomic organization of Tetranychus urticae-associated narnavirus is also indicated. Grey arrows: putative NS.

Finally, the Tetranychus urticae-associated narnavirus, present in 0.15% and 0.2% of reads in P and F populations, respectively, was the only one found in this study that historically belongs to a family of fungi-infecting viruses. Although very little is known regarding these viruses, the phylogenetic position of its polymerase protein within a clade including associated narnaviruses, as well as members of the *Ourmiavirus* genus, suggests that it might represent a new species-level lineage according to the ICTV species demarcation threshold (<50% of protein sequence identity as compared to the closest relative) (Figure 6, Table 1).

### 3.2. Genomic and Transcriptomic Database Screening

To gain insights into the diversity and the distribution of viruses in *T. urticae*, we further screened mite genomic and transcriptomic datasets using all ten viruses that were identified in this study as queries. Our search highlighted that *T. urticae* transcriptomes contained sequences that displayed >95% of nucleotidic identity to Aphis glycines virus 1 and Tetranychus urticae-associated picorna-like virus 1 (Table 2). Interestingly, one sequence that was related to an ambidensovirus was found in the genome of *T. urticae*, but this sequence was different from TuaDV (70% of nucleotidic identity with a sequence of 536 nt length). No sequence corresponding to TuaDV was found in any of the transcriptomes that were analyzed, suggesting that this virus might correspond to a non-endogenized virus, whose origin remains to be clarified.

In addition to these viruses, we found in *T. urticae* genomes and transcriptomes, 16 sequences belonging to large viruses, including giant viruses (*Mimiviridae*, *Phycodnaviridae*, *Poxviridae*, and *Baculoviridae*). These sequences were not found in the viromes that we generated in this study (i.e., using VANA based method) and we cannot exclude that the sequences corresponding to this large dsDNA virus might have a cellular rather than a viral origin. Eight sequences that were related to *Rhabdoviridae* were found in the *T. urticae* genome, which all corresponded to the nucleoprotein N, and we also found these sequences in the EST transcriptome (six sequences > 95% of nucleotide identity). Last, we also found sequences that were related to plant viruses in the TSA transcriptome of *T. urticae* that might originate from diet contamination. One transcriptomic sequence (accession number GW017620.1) matched with the Tetranychus urticae-associated nodavirus segment B1, although its small size did not allow its for assignation with high confidence (Table 2).

### 3.3. Characterization of a Novel Densovirus in Spider-Mites

As pointed out above, the size of the densovirus contig found in mites populations was about 3.4 kb, which was much smaller than genomes characterized so far in the *Ambidensovirus* genus, which, when excluding the terminal repeats (ITRs), is around 4 kb for the Acheta domesticus mini ambidensovirus and up to 5kb for Lepidopteran ambidensoviruses [34]. Ambidensoviruses usually display a single ORF encoding for one to four structural proteins (VP1–4) that are produced by splicing or leaky scanning, and three ORFs encoding for non-structural (NS) proteins. Viruses in the *Parvoviridae* family are characterized by two typical domains: i) a phospholipase A2 (PLA2) motif located in VP1 of most parvoviruses, including in all species described so far in the *Ambidensovirus* genus; and, ii) a Super Family 3 (SF3) helicase domain that is located in the NS1 protein and common to all parvoviruses [35].

**Figure 7:**
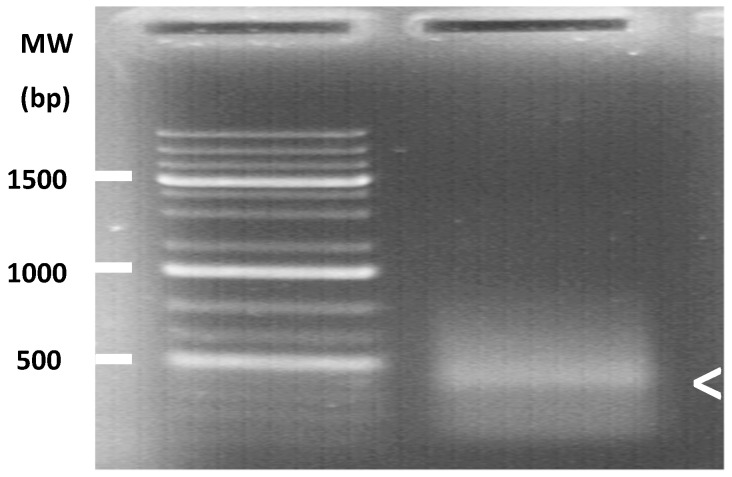
Identification of the transcription starting site of ORF2 (*ns* gene) of the TuaDV by 5’ RACE PCR. Agarose gel showed a major amplicon at ~500 bp (white arrowhead). The amplicon was sequenced to recover the 5’ end sequence.

The contig corresponding to the TuaDV genome predicted three to four open reading frames (ORFs), one encoding a typical VP protein (ORF1), and two to three putative ORFs encoding NS proteins (ORF2–4) (Figure 2). 5’ and 3’ RACEs further verified the 5’ and 3’ ends of the viral genome and we performed overlapping PCRs with specific primers and sequenced the amplicons using the Sanger method (Figure 7). These results further confirmed that both of the populations shared the same TuaDV genomic sequence and that both expressed viral genes.

We did not obtain any complementary sequence for ORF1 (VP), which thus predicted a 505-amino acids (aa) protein of 55 kDa that lacked the typical PLA2 motif that is common to all ambidensoviruses described so far. Concerning the reverse strand, we obtained the complete coding sequence of the NS ORFs with a predicted ORF2 (NS1) sequence of 354 aa protein of 39 kDa, harboring the typical SF3 domain, while a predicted ORF3 (NS2) has a 273 aa protein and a molecular weight of 30 kDa. In addition, a small ORF4 (95 aa) was predicted and it might encode for a putative 10 kDa NS3 protein. With such a compact genome, this virus would be the smallest densovirus that is described so far, including the Acheta domesticus mini ambidensovirus, with both viruses having sizes that are comparable to species from the *Iteradensovirus* genus. The closest relative of TuaDV was the Lupine feces-associated densovirus 2 (accession number KY214445.1), which shares 30% identity with NS1 and 31% identity with VP [36]. The putative NS2 of these viruses shared 25% identity. If correct, these NS predicted ORFs would have an ORF3 initiating upstream ORF2, which is unusual among ambidensoviruses. Based on the current species demarcation criteria that were used by the ICTV, all of these features suggest that TuaDV is a new mini ambidensovirus species among the *Ambidensovirus* genus that shares little sequence identity with the Acheta domesticus mini ambidensovirus.

Given the theoretical rapid evolution of ssDNA viruses [37], the fact that mite populations with different origins and rearing history share identical virus sequences more likely suggests that contamination occurred between laboratories, due to mites and material exchange, which is also supported by the introduction of the Almeria population in the Montpellier laboratory at the time.

### 3.4. TuaDV Prevalence and Diversity in Mite Populations

**Figure 8:**
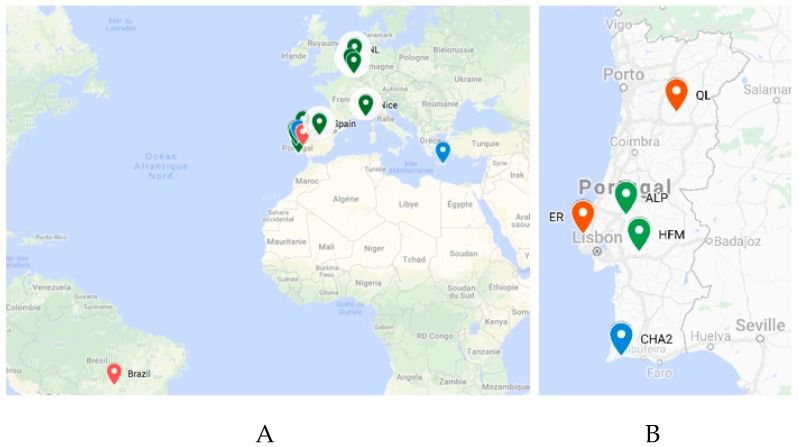
Map representing the origin of spider mites’ populations worldwide (**A**) and in Portugal (boxed) (**B**) where the prevalence of TuaDV has been assayed by PCR and sequenced (full or partial genome) and in Portugal (**B**). *T. urticae* (green), *T. cinnabarinus* (blue), and *T. evansi* (red). Maps were made from maps.google.com.

To better understand the origin of the TuaDV that infected the P and F reared populations (so-called here after TuaDV_Rearing (R) to discriminate from _Field populations), we investigated its prevalence and diversity in different *Tetranychus* species with different origins. Twenty-seven samples from three species of *Tetranychus* (*T. urticae*, *T. cinnabarinus*, and *T. evansi*) were collected from various fields in Portugal and from rearing facilities worldwide (France, Brazil, Netherlands, Belgium, and Crete), representing 19 and eight samples, respectively (Figure 8). Interestingly, the genome of the Lupine feces densovirus 2 was recently discovered by metagenomics from feces of Iberian wolves that were sampled in Portugal (South Douro region), which suggests that distant relatives of TuaDV are present in the environment, at least on the Iberian Peninsula.

TuaDV presence and its genomic sequences in each population were confirmed by conventional PCRs using various sets of primers and sequencing (see Materials & Methods section). Note that each sample corresponds to a pool of mites from the same species that were collected in the same field/rearing. Amplicons were obtained with all sets of primers and they were of the expected size, for all samples, thus indicating 100% prevalence in the samples, and suggests a wide prevalence of TuaDV in spider mite populations, both in natural environments and from all of the rearing facilities tested. However, we cannot determine whether all of the individual mites were infected or few were highly infected.

To investigate the phylogenetic relationship between the viruses, we sequenced TuaDV amplicons from 11 selected samples, i.e., four *T. urticae* (a second sample from France, Belgium, Netherlands, and Portugal), four *T. cinnabarinus* (Portugal), and three *T. evansi* (Portugal and Brazil); for each species, one sample corresponds to mites that were collected from the field. We used various sets of primers in order to cover the full-length genome or the ORF2 (NS1 gene) only (Table S1).

We reconstructed the TuaDV full length genomes from three species that were recently collected in Portugal by sequence assembly and alignments. The recovered genomes displayed a high identity score (Table 3), with the TuaDV that was isolated from *T. evansi* being the most distantly related (81% identity with the original TuaDV_R genome; 86% when considering only the identity of NS1); although, these viruses belong to the same species according to the species demarcation criteria of the ICTV. The TuaDV genomes that were sequenced from *T. urticae* and *T. cinnabarinus* were both similar to the initial TuaDV_R (99.8%; 100% identity of NS1), with only three and five synonymous substitutions between TuaDV_R and TuaDV from *T. cinnbarinus* and *T. urticae* from natural populations, respectively; the virus from the two natural populations differed by five substitutions, which further suggests that TuaDV_R originates from *T. cinnabarinus*. This relatedness is congruent with the phylogenetic position of *T. urticae* and *T. cinnabarinus*, often considered as two morphs of the same species, with *T. evansi* being more distantly related [19,20]. Moreover, it suggests that the infection of the population reared in Portugal originated from the Almeria population introduced in the rearing three years ago (see methods section).

To better assess the prevalence and origin of the TuaDV, we partially sequenced ORF2 from 10 other spider mite samples that originated from natural and rearing populations worldwide. The results also revealed high identity scores between all of the populations, with the highest being within species (100% identity at the nucleotidic level) independently of their origin, i.e., from natural populations and rearings in Portugal.

Altogether, these results suggest that TuaDV is prevalent in natural populations of *Tetranychus* in Portugal and it displays a genetic diversity that likely correlates with the mite phylogeny. The poor genetic diversity that exists between the *T. urticae* laboratory and natural populations, with the exception of the Nice population, is striking and it suggests that infections of laboratory populations occurred in Portugal from wild populations and then spread between rearing facilities worldwide with material exchanges between laboratories. A broader geographical sampling of natural populations, including more *Tetranychus* species, needs to be performed in order to test this hypothesis and evaluate the diversity of TuaDV worldwide.

### 3.5. TuaDV Can be Vertically Transmitted and Circulate in Planta

In the search for the TuaDV route(s) of transmission, first we assayed TuaDV prevalence in individual mites using PCR. We found that 90% (18/20) of the individuals from the population HH4 tested were positive for TuaDV. Due to this high prevalence and the absence of an uninfected population, we did not test for horizontal transmission. We tested vertical transmission (from mothers to offspring), from 10 females from the HH4 population that all layed eggs and then tested 10 offspring per positive female. We found that seven females were positive to TuaDV, out of which four females had positive offspring, with levels of vertical transmission that greatly differed between females. Mean levels of vertical transmission was estimated at 28% (± 0.9 SE), and it was highly variable between females with 8/10 for one female and 5/10 for the 3 others (versus 0/10 for females where no vertical transmission was detected). Such variability between offspring probably revealed quantitative variability in the vertical transmission of TuaDV. Although, we cannot exclude to have underestimated the vertical transmission due to the limit of the detection method.

**Figure 9:**
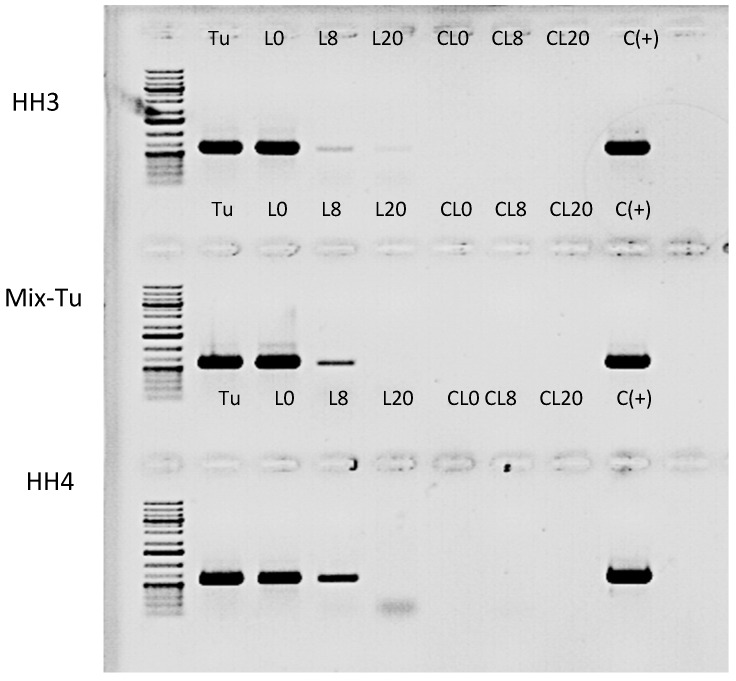
Transmission of TuaDV in plants. PCR from total DNA (100ng) extracted from mites, their isolated feeding leaf (L0) and two non-infested leaves at 8 and 20 cm distance (L8 and L20 respectively). Leaves from non-infested plants (CL0, CL8, and CL20) were used as negative controls, while mites were used as positive controls. Three independent F-populations were assayed (HH3, Mix-Tu, and HH4).

Fecal-oral contamination is a common route of horizontal transmission. Interestingly, an aphid densovirus (MpDV) has been shown to be injected into plants by aphids and circulate *in planta*, which thus participate to its horizontal transmission to conspecifics [21]. Unlike aphids, mites used their stylets to pierce leaf mesophyll cells, where they then inject saliva and suck the cell cytoplasm. Mites are not considered to be vectors, although their feeding behavior injures their host, which can cause the transmission of viruses, in a way that is similar the mite *Varroa destructor* transmit viruses to bees [16]. To test whether TuaDV could be transmitted to the plant during mite feeding, we set up an infestation assay on bean plants (see method section) and assayed virus transmission to plant leaves by PCR. The results obtained from all pairs of primers showed that the leaves on which mites were reared on (L0) were all positive for the three populations tested. This result showed that infected mites have contaminated leaf surfaces, which further suggests that feces and/or saliva are a source of virus (Figure 9). Interestingly, the distant leaves (L8 and L20) were also positive, although the levels decreased with the distance from the primary leaf (L0), suggesting that the virus could circulate within the plant without replicating. Control leaves from the non-infested plants (no mites) and negative controls with no template were negative. We next wanted to quantify the likelihood of TuaDV transmission from the mite to the plant and between the leaves of different distances. Quantification of TuaDV from mites and leaves was performed using a pair of specific primers in ORF2 and a standard curve that was established from serial dilutions of a construct containing the Junonia coenia ambidensovirus genome as a proxy for TuaDV genome quantification. Negative controls (leaves with not mites) displayed Ct values that were similar to negative controls with no template. We estimated that the load of TuaDV per mite was 7.10^6^ veg (± 8.10^5^ SE), and we found 2.10^7^ veg (± 4.10^6^ SE) on L0, which represents the viral shedding from 50 mites for five days. Thus, viral shedding per mite with saliva and feces was estimated at 9.10^4^ veg (± 1. 10^4^ SE)/day, which represents 1.5% (± 0.37 SE) of the total amount of virus estimated per mite. Concerning leaves at 8 (L8) and 20 cm (L20) distance from L0, we found 2.10^4^ veg (± 7.10^3^ SE) and 3500 veg/leaf (± 3100 SE) in L8 and L20, respectively, with an increasing variation between experiments when increasing the distance from L0. Based on these results, we estimated that 0.08 % (± 0.02 SE) of TuaDV contaminating L0 was inoculated into the plant vascular system and recovered in L8, and representing 0.017% (± 0.016 SE) in L20. Levels of virus declined with distance from the infested leaf (F _2, 10_ = 124.08, *p* < 0.0001), but there was no different between the different populations (F _2, 3_ = 3.28, *p* = 0.1757).

Altogether, these results showed that *T. urticae* could vertically and horizontally transmit TuaDV. Vertical transmission varies greatly between females, suggesting an important variation in the amount of virus that can be transmitted. Horizontal transmission occurred through the contamination of food (i.e., plant tissues) from virus shedding with feces and/or saliva. In addition, mites inoculate TuaDV to the plant vascular system, which then can probably circulate from cell-to-cell through plasmodesmata because of its small size (~20nm), to systemically reach distant leaves.

## 4. Discussion

In the work that is presented here, we used a viral metagenomic approach to explore the diversity of viruses that are associated with the spider mite *Tetranychus urticae*, a small arthropod that has long been neglected by virologists. We showed that several viruses simultaneously circulate in mites, including a panel of eight putative virus species that belong to small, non-enveloped viruses with a RNA genome, and one ssDNA virus with a predicted ambisense genomic organization and belonging to the *Densovirinae* sub-family [34]. This novel densovirus displays two uncommon features among ambidensoviruses, i.e., the most compact genome identified so far, all densoviruses included, and the absence of the PLA2 motif in the major capsid protein.

An unexpected result was that two mite laboratory populations with different origins shared a set of five viruses, representing 5 taxa. If we independently consider these viral taxa replicate, then the probability is low that five phylogenetically distant viruses were found together in two independent populations (although composed of the same host species) just by chance. Thus, the most parsimonious explanation is to consider that a cross contamination occurred between laboratories, especially when considering the absence of diversity between the common virus genotypes. One possibility is that the Almeria population of *T. cinnabarinus*, which was introduced three years ago in the Portuguese rearing, was at the origin of the contamination of the F population, as it was also maintained in Montpellier laboratory at the time the F-population was sampled.

A second unexpected result was to find that three years after we identified TuaDV (which represents more than 70 mites generations), nearly identical virus genotypes were still found to be circulating in both rearings, while considering the expected occurrence of sequence change in viruses due to error-prone replication (particularly high for RNA and ssDNA viruses [37]). One possibility could be that sequence change of virus genomes was slower than expected. In support of that hypothesis, we found that TuaDV genomic sequence from rearings differed by a few substitutions (3 to 5) from the sequences that were found recently in the natural populations of *T. urticae* and *T. cinnabarinus* sampled in Portugal and in different rearing facilities worldwide. Although the level of relatedness was high, we did observe slight differences, indicating that TuaDVs from the Netherlands (Santpoort) and Belgium (Ghent) were more closely related than they were to the samples from Portugal, and the TuaDV from Greece (Crete) was almost identical to the TuaDV from Portugal (99 to 100%). When considering that the Greek population is actually composed of a mix of populations from different origins, this suggests that a cross contamination probably occurred between the laboratories. More interestingly, we found that the TuaDV from the French population of *T. urticae* that originates from Nice-Valbonne was more distantly related to the other strains (88% with the ORF2 of TuaDV_R versus 94 to 100% that are shared by the other *T. urticae* samples). This population was collected in the Nice area 3–5 years ago and then reared there since then, without any exchange with the other facilities of this study (M. Ferrero, personal communication). This suggests that several strains of TuaDV probably circulate in natural populations. We speculate that TuaDV might be under stabilizing selection when circulating in *T. urticae* and *T. cinnabarinus*, that is that most new mutations would be deleterious and thus be quickly removed from the viral populations [38,39]. Moreover, the high identity score of the TuaDV genome found in two ”sister” species, *T. cinnabarinus* and *T. urticae*, and the distance with TuaDV from *T. evansi* further supports that divergence and evolution have co-occurred with their hosts’ evolution.

The third unexpected result was the discovery in mites viromes of contigs corresponding to three unclassified picorna-like related to Aphis glycines virus 1, one of them sharing 99% nucleotidic identity with the virus that was discovered in aphids at the University of Illinois and published in Genebank in 2013 (#KF360262), the two others being more distantly related (Table 1). Like for TuaDV, such sequence conservation was striking when considering the time and the geographic distance between sampling but also the phylogenetic distance between aphids and mites. The constraints of the host immune system are among mechanisms conditioning virus changes for adaptation. Interestingly, mites and aphids share a genome that has lost canonical components of the immune system [40]. We speculate that such feature may contribute to provide tolerant environments to viruses. Although, the mechanism remains to be addressed.

Most of the virus genotypes that were found in this study were classified within arthropod-infecting taxa and/or their closest phylogenetic taxa were associated with arthropods. The phylogenetic trees that we obtained for each virus highlighted the poor knowledge that we have on arthropod viruses [41,42,43], with viruses often being located at the base of the phylogenetic trees of the largest taxa, such as for *Parvoviridae* and *Picornavirales*, or grouped into poorly documented viral families, which indicates that a wealth of viruses from these groups remain to be discovered. The set of viruses that we found in this study mostly corresponds to small, non-enveloped viruses, which contrasts with sequences that are found in nucleotidic databases and corresponding to large viruses. The under-representation of large viruses in viromes prepared with the VANA method may result from a technical bias. Indeed, the filtration step that is used in this method to prepare viral particles could eliminate large viruses [44]. This observation thus pinpoints the need to combine approaches to get an exhaustive view of virus diversity that is associated with arthropods.

In natural or experimental populations, mite density can vary due to variation in mortality levels, which can occur without the knowledge of the causal agent(s)/conditions. Whether and how TuaDV affects the mites phenotype remains to be experimentally addressed. As the level of vertical transmission is relatively low, the establishment of TuaDV-free lines to investigate the role of TuaDV by experimental infection is made possible. Interestingly, persistent densoviruses can be found in aphids where they have been shown to confer protection against secondary infections by other pathogens, including viruses and bacteria [45,46]. Unlike those observed for densoviruses that were discovered in aphids, we could not find evidence of virus genomic integration in the mite genome, suggesting that the TuaDV could correspond to an extant virus [4,47]. However, we cannot exclude that viral sequences were cleaned off during *T. urticae* genome assembly [8].

It is becoming clear that both multi-infections and persistent viruses should be considered in order to better understand the phenotypic outcome of infections and the pathogenicity of specific strains that can change their life cycle, from pathogenic to persistent [48]. Densovirus persistent infections are likely common in aphids, and several densoviruses have been described, with one being transmitted through the plant [21,45,47]. Although we need to improve the viral loads quantification method, our results suggest that potentially large amount of TuaDV can contaminate plants when considering the high densities that mites colonies can reach. In a previous study, we described a new densovirus found from viral metagenomics from sea barley (*Hordeum marinum*), probably contaminating/circulating in the plant as well, since the virus was recovered from different leaves and was not associated with any arthropods host [49]. We hypothesized that plants participate to TuaDV horizontal transmission by concentrating viral particles on leaves, thus favoring the spreading of infection in mite populations. Furthermore, the systemic inoculation of viral particles in the plant vascular system could also provide the virus a protective niche against abiotic factors (particularly ultraviolet radiation from the sun) that can be detrimental for viral particles. Interestingly, our results also suggest that the plant might also mediate infections with a panel of arthropod-infecting viruses.

We hypothesized that virus abundance and/or transmission routes might have been selected for virus communities that occur as a unit within their host populations. Indeed, we find that five viruses co-occur and can be maintained in two separate laboratory populations for three years, even if the source is due to a recent contamination. Our results suggest that transmission can occur by vertical and horizontal routes, including via the host plant, leading to a wide prevalence in their host populations. This begs the question as to whether all of these viruses can occur in a multiple infection within single individual hosts or if they never co-infect but separately circulate in mite populations (i.e., one virus preventing the infection by others). Further investigation is required to evaluate whether these viruses interact in a synergic, antagonist, or neutral manner.

In addition to sharing common ecosystems and similar immunologic environments, mites and aphids also share diverse endosymbionts that may provide protection in compensation [40,50]. Whether and how viruses could also provide protection against immunological stress remain to be addressed. Experimental mite colonies could provide a powerful system to combine descriptive and manipulative experiments to test for virus pathogenicity in individual hosts; and their dynamics (prevalence and persistence), and evolution in host populations.

## Figures and Tables

**Table 1:** Description of the fourteen virus contigs found in *T. urticae* viromes and protein identity comparison with their closest relatives.

Baltimore Classification	Viral Family	Viral Contig	Contig Length (nt)	Putative Protein	Best Hit (BLASTx)	Accession Number	Protein Identity (%)
ssDNA	*Parvoviridae*	Tetranychus urticae-associated ambidensovirus	3411	polymerase (NS1)	*Lupine feces-associated densovirus 2*	ASM93489.1	39%
				capsid		ASM93488.1	30%
dsRNA	*Birnaviridae*	Tetranychus urticae-associated birnavirus Segment A	2899	polymerase	*Infectious bursal disease virus*	AAS10174.1	30%
		Tetranychus urticae-associated birnavirus Segment B	2501	polyprotein	*Blotched snakehead virus*	YP_052864.1	33%
ss+RNA	*Nodaviridae*	Tetranychus urticae-associated nodavirus Segment A 1	3230	polymerase	*Hubei noda-like virus 9*	APG76321.1	58%
		Tetranychus urticae-associated nodavirus Segment A 2	2538	polymerase	*Hubei noda-like virus 8*	YP_009337881.1	62%
		Tetranychus urticae-associated nodavirus Segment B 1	1661	capsid	*Hubei noda-like virus 9*	YP_009337880.1	21%
		Tetranychus urticae-associated nodavirus Segment B 2	1658	capsid			53%
		Tetranychus urticae-associated nodavirus Segment B 3	1570	capsid			58%
	*Dicistroviridae*	Tetranychus urticae-associated dicistrovirus 1	8290	polymerase	*Beihai picorna-like virus 70*	APG78061.1	24%
				capsid		APG78062.1	20%
		Tetranychus urticae-associated dicistrovirus 2	8449	polymerase		APG78061.1	23%
				capsid		APG78062.1	20%
	Unclassified *Picornavirales*	Aphis glycines virus 1	8592	polymerase	*Aphis glycines virus 1*	AHC72013.1	96%
				capsid		AHC72012.1	99%
		Tetranychus urticae-associated picorna-like virus 1	8151	polymerase		AHC72013.1	71%
				capsid		AHC72012.1	75%
		Tetranychus urticae-associated picorna-like virus 2	6432	polypotein	*Hubei picorna-like virus 80*	YP_009337381.1	53%
	*Narnaviridae*	Tetranychus urticae-associated narnavirus	2481	polymerase	*Hubei narna-like virus 3*	YP_009337787.1	45%

**Table 2:** Summary of the number of sequences found in the *Tetranychus urticae* genomic and/or transcriptomic databases that display homologies with viral species discovered in the viromes generated in this study.

Viral Contig	Contig Length (nt)	ref_seq Genomics	EST	TSA
Tetranychus urticae-associated ambidensovirus	3411	0	0	0
Tetranychus urticae-associated birnavirus Segment A	2899	0	0	0
Tetranychus urticae-associated birnavirus Segment B	2501	0	0	0
Tetranychus urticae-associated nodavirus Segment A 1	3230	0	0	0
Tetranychus urticae-associated nodavirus Segment A 2	2538	0	0	0
Tetranychus urticae-associated nodavirus Segment B 1	1661	0	**1**	0
Tetranychus urticae-associated nodavirus Segment B 2	1658	0	0	0
Tetranychus urticae associated nodavirus Segment B 3	1570	0	0	0
Tetranychus urticae associated dicistrovirus 1	8290	0	0	0
Tetranychus urticae associated dicistrovirus 2	8449	0	0	0
Aphis glycines virus 1	8592	0	**105**	0
Tetranychus urticae associated picorna-like virus 1	8151	0	0	**14**
Tetranychus urticae associated picorna-like virus 2	6432	0	0	0
Tetranychus urticae associated narnavirus	2481	0	0	0

**Table:** 

	*T. cinnabarinus*	*T. evansi*	*T. urticae*_Field	*T. urticae*_R (TuaDV)
*T. cinnabarinus*		(79.0; 79.6)%	(99.8; 99.4)%	(99.8; 100)%
*T. evansi*	(79.0; 79.6)%		(79.0; 79.6)%	(78.9; 79.6)%
*T. urticae*_Field	(99.8; 99.4)%	(79.0; 79.6)%		(99.6; 99.4)%
*T. urticae*_R (TuaDV)	(99.8; 100)%	(78.9; 79.6)%	(99.6; 99,4)%	

**Table:** 

	*T. evansi*	*T. urticae*_Field	*T. urticae*_R (TuaDV)
*T. evansi*		(83.3; 86.0)%	(83.2; 86.5)%
*T. urticae*_Field	(83.3; 86.0)%		(99.9; 99.7)%
*T. urticae*_R (TuaDV)	(83.2; 86.5)%	(99.9; 99.7)%	

## Supplementary Materials

The following are available online at https://www.mdpi.com/1999-4915/11/3/233/s1, Table S1: *Tetranychus* species screened for the presence of TuaDV, Table S2: Sequences of TuaDV primers.
