# Supplementary material for: A New Prevalent Densovirus Discovered in Acari. Insight from Metagenomics in Viral Communities Associated with Two-Spotted Mite (Tetranychus urticae) Populations
Source: Viruses. 2019 Mar 7;11(3):233. doi: 10.3390/v11030233 (PMC6466187; doi:10.3390/v11030233)
Supplement: Supplementary file 1 [file viruses-11-00233-s001.pdf]

Table S1: *Tetranychus* species screened for the presence of TuaDV. In yellow and in green: samples that have been fully and partially sequenced respectively. * represents species from rearings for more than one year.

**General Information**			**Lab/Field information**				
**Species**	**Population**	**Form**	**Country**	**Sampling location**	**Sampling date**	**Initial number of mites**	**plant**
*T. urticae**	R5	Green	Netherlands	Netherlands	2009	300	Bean
*T. urticae**	Santpoort 2	Green	Netherlands	Santpoort	2001	~250	Bean
*T. urticae**	BE	Green	Belgium	Ghent	NA	NA	Bean
*T. urticae**	Nice-Valbonne	Green	France	Nice-Valbonne	2012	NA	Bean
T. urticae	HFM	Green	Portugal	Herdade do Freixo do Meio - Montemor-o-Novo	2017	80	Tomato
T. urticae	MON	Green	Portugal	Quinta Vidigal - Montemor-o-novo	2017	150	Tomato
T. urticae	ALP	Green	Portugal	Agrial - Alpiarça	2017	90	Tomato
T. urticae	DEF	Green	Portugal	Alvalade - Lisbon	2017	300	Tomato
*T. urticae**	AMP	Red	Portugal	Aldeia da Mata Pequena	2017	60	Bean
T. urticae	AMP.2	Red	Portugal	Aldeia da Mata Pequena	2017	30	Bean
*T. cinnabarinus**	SB9	Red	Crete	MIX	2006	Selection x females	Bean
*T. cinnabarinus**	SB9.2	Red	Crete	MIX	2006	100	Bean
T. cinnabarinus	CHA2	Red	Portugal	Lagos	2017	50	Tomato
T. cinnabarinus	BF	Red	Portugal	Lourinhã	2017	50	Tomato
T. cinnabarinus	PBT	Red	Portugal	Gradil (monte gordo)	2017	70	Tomato
T. cinnabarinus	LIM	Red	Portugal	Lagoa	2017	500	Tomato
T. cinnabarinus	6M2	Red	Portugal	Chamusca	2017	100	Tomato
*T. evansi**	BR	T2	Brazil	unknown	2002	300	Tomato
*T. evansi**	GH	T1	Portugal	Uni Lisbon	NA	80	Tomato

*T. evansi**	QL	T1	Portugal	Quinta das Lameiras	2013	500	Tomato
T. evansi	CG	T2	Portugal	C7 (campo grande) - Lisbon	2017	320	Tomato
T. evansi	VC	T2	Portugal	Parque urbano do vale de Chelas - Lisbon	2017	80	Tomato
T. evansi	ER	T2	Portugal	Ericeira - Ericeira	2017	100	Tomato
T. evansi	QG	T1	Portugal	Quinta da Granja (Colombo) - Lisbon	2017	180	Tomato
T. evansi	PBS	T1	Portugal	Parque da Bensaude (Laranjeiras) - Lisbon	2017	150	Tomato
T. evansi	6M1	T1	Portugal	Lagos	2017	400	Tomato
T. evansi	VIT	T1	Portugal	Vitacress - Alcochete	2017	300	Tomato

Table S2: Sequences of TuaDV primers.

Primer name	Primer sequence	Primer length
20 F	GAGCCTTCAACTAGCACAGC	20
61 F	ACCTGGTGGAAGCTTACGAC	20
639 R	GGCATAATTGAAGGCTCGTTCC	22
556 F	AAACTTTGTACGTGGACAAAACT	23
1,125 F	AGCAGCATTAGATTGAACAGGT	24
1,203 F	TCAACTTGGACAATCTCAACAACC	22
1,793 R	ACACTGGTTCAAATAGCTTATGCA	24
2,070 R	TCAAATCTACACGGCTGGTT	20
1,230 R	TATTGTAATTATCAATTTCA	20
